# Mechanism of water-stress induced cavitation in conifers: bordered pit structure and function support the hypothesis of seal capillary-seeding

**DOI:** 10.1111/j.1365-3040.2010.02208.x

**Published:** 2010-12

**Authors:** SYLVAIN DELZON, CYRIL DOUTHE, ANNA SALA, HERVE COCHARD

**Affiliations:** 1 University of Bordeaux, UMR 1202 BIOGECOF-33405 Talence, France; 2 INRA, UMR 1202 BIOGECO69 route d'Arcachon, 33612 Cestas, France; 3 Division of Biological Sciences, The University of Montana, MissoulaMT 59812, USA; 4 INRA, UMR 547 PIAFF-63100 Clermont-Ferrand, France

**Keywords:** bordered pit, cavitation, conifers, drought, torus, xylem

## Abstract

Resistance to water-stress induced cavitation is an important indicator of drought tolerance in woody species and is known to be intimately linked to the anatomy of the xylem. However, the actual mechanical properties of the pit membrane are not well known and the exact mode of air-seeding by which cavitation occurs is still uncertain. We examined the relationship between cavitation resistance and bordered pit structure and function in 40 coniferous species. Xylem pressure inducing 50% loss of hydraulic conductance (*P*_50_, a proxy for cavitation resistance) varied widely among species, from −2.9 to −11.3 MPa. The valve effect of the pit membrane, measured as a function of margo flexibility and torus overlap, explained more variation in cavitation-resistance than simple anatomical traits such as pit membrane, pit aperture or torus size. Highly cavitation resistant species exhibited both a high flexibility of the margo and a large overlap between the torus and the pit aperture, allowing the torus to tightly seal the pit aperture. Our results support the hypothesis of seal capillary-seeding as the most likely mode of air-seeding, and suggest that the adhesion of the torus to the pit border may be the main determinant of cavitation resistance in conifers.

## INTRODUCTION

Conifers are a very important component of terrestrial ecosystems and occur from dry woodlands to the vast boreal forests. Despite their homogenous wood (with only tracheids), conifers exhibit considerable variation in hydraulic properties; for instance, variation among and within conifer species has been documented in hydraulic conductivity (Domec & Gartner 2002), tracheid size (Pittermann *et al*. 2006a), wood density (Pittermann *et al*. 2006b) and cavitation resistance (Pinol & Sala 2000; Maherali, Pockman & Jackson 2004; Martinez-Vilalta, Sala & Pinol 2004; Brodribb & Cochard 2009).

Because the ascent of water within the secondary xylem of trees involves crossing inter-conduit pit pores, pit structure and function are very important in controlling water movement. Several studies have suggested that resistance to drought-induced embolism is proportional to the ability of pits to prevent air-seeding (Jarbeau, Ewers & Davis 1995; Hacke, Sperry & Pittermann 2004). Indeed, a major cause of xylem cavitation appears to be air leakage from embolized conduits through inter-conduit pits (Crombie, Hipkins & Milburn 1985; Cochard, Cruizat & Tyree 1992; Jarbeau *et al*. 1995). According to this air-seeding hypothesis, cavitation occurs when air bubbles are sucked from a non-functional air filled conduit into a functional conduit through the inter-conduit pits. Because the ability of water to resist air entry through cell wall pores depends on water capillary forces, which, in turn, depend on the diameter of the pore, vulnerability to cavitation increases with increases of the porosity of the pit membranes. Accordingly, variations in xylem anatomical traits among species have been related to cavitation resistance (Hacke *et al*. 2004; Domec, Lachenbruch & Meinzer 2006). In angiosperms, cavitation resistance depends on the size of the largest pores in the pit membranes. Accordingly, greater cell expansion during vessel maturation often results in wider pit area, larger membrane pores and, consequently, lower cavitation resistance (Wheeler *et al*. 2005; Christman, Sperry & Adler 2009).

**Figure 1:**
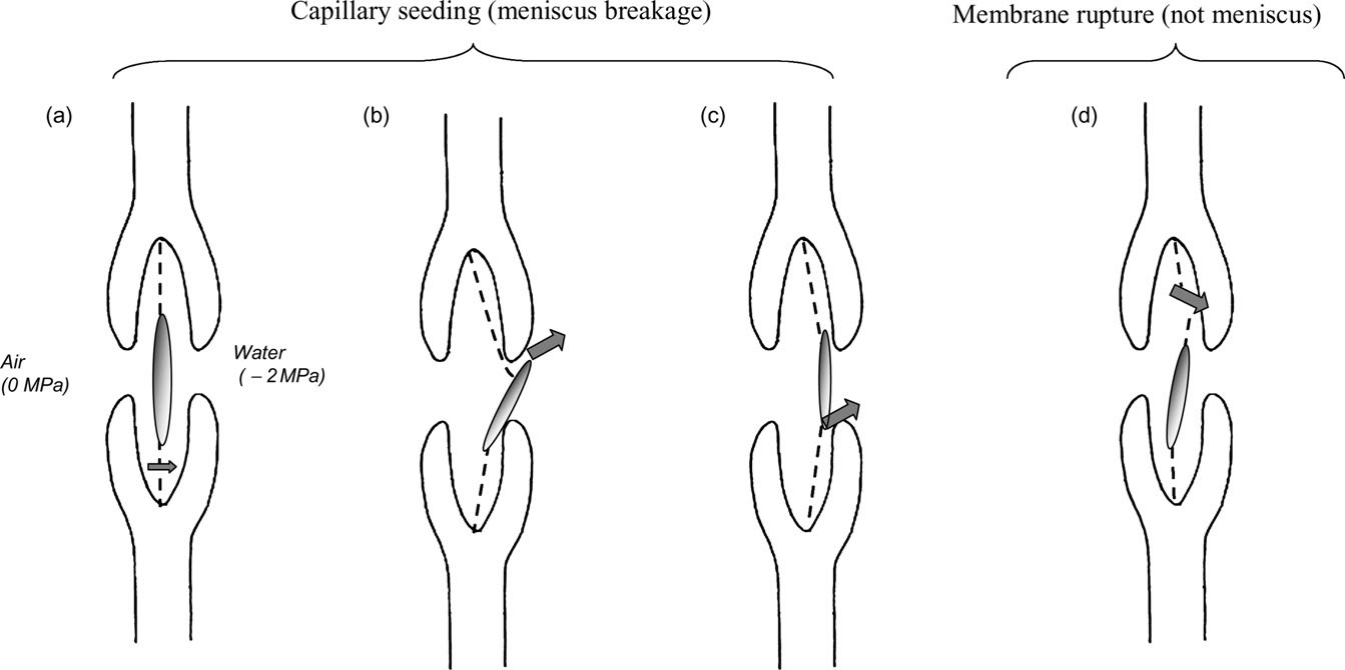
Different hypotheses of air-seeding through a conifer bordered pit membrane: (a) margo capillary-seeding, by capillary rupture of an air/water meniscus through pores in the margo when torus aspiration does not occur; (b) margo stretch-seeding, by elastic stretching allowing the torus to be pulled out through the pit aperture or through membrane slippage that allows the torus to move off-center; (c) seal capillary-seeding, when the torus is not tightly sealed against the pit border (weak aspiration or poor air tightness of torus/pit aperture interface); and (d) margo rupture-seeding, by membrane breakage.

On the other hand, the inter-tracheid pits of gymnosperms are quite different in structure and function from inter-vessel angiosperm pits, and their roles in cavitation resistance is poorly understood (but see Domec *et al*. 2006; Hacke & Jansen 2009). Gymnosperm inter-tracheid pits are complex and quite specialized (Bailey 1916; Gregory & Petty 1973), and usually consist of circular bordered pits, where the secondary wall arches over the torus-margo pit membrane (Fig. 1; Sachs 1963; Bauch, Liese & Schultze 1972; Hacke *et al*. 2004; Pittermann *et al*. 2005). Pit membrane porosity, including that of the torus and margo of conifers, appears to be involved in air-seeding events and is thought to play the most important role in cavitation resistance (Sperry & Tyree 1990; Tyree & Zimmermann 2002). We now know that the essentially impermeable torus has the capacity to exert a valve effect by sealing the pit aperture (Bailey 1916; Pittermann *et al*. 2005). When the pressure difference between the functional and embolized tracheids is high enough, the flexible margo allows the torus to be displaced against the pit border, thereby sealing the pit aperture and preventing subsequent air seeding.

**Figure 2:**
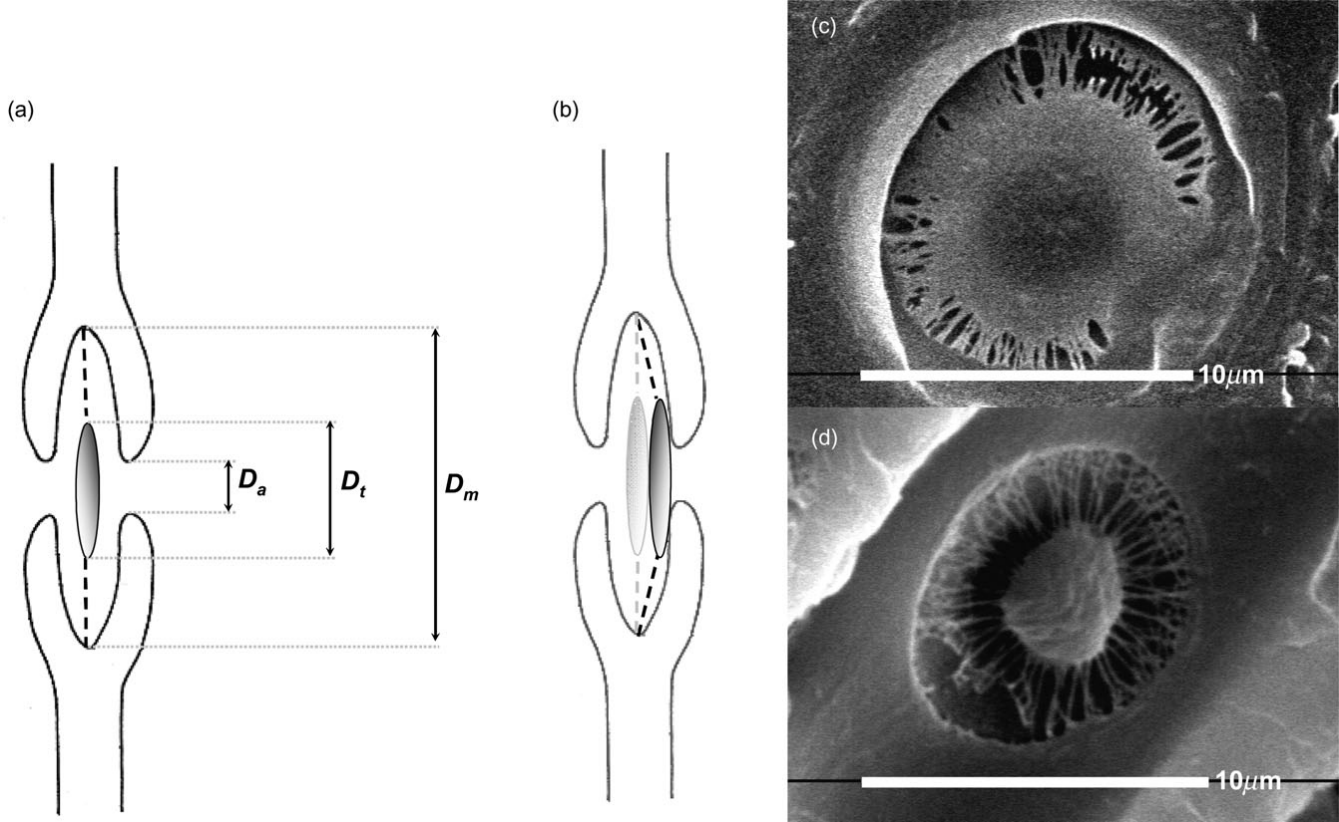
Schematic representation of a bordered pit membrane of conifer on the left. In the first inset (a), important features are represented, namely pit aperture diameter (*D*_a_), torus diameter (*D*_t_) and pit membrane diameter (*D*_m_). The second inset (b) represents the torus valve effect (*V*_ef_, i.e. pit aspiration and torus overlap between torus and pit aperture). Torus is both represented in its unaspirated (grey) and aspirated positions (black). On the right, comparison of bordered pit membrane anatomy between a species vulnerable to cavitation (*P. albicaulis*, c) and a species resistant to cavitation (*C. glabra*, d). White bars represent 10 *µ*m.

How does cavitation occur with such a pit membrane structure? In a recent overview, Cochard (2006) proposed four different mechanisms of air-seeding in conifers. However, it is currently unknown whether as a general rule, air seeding actually occurs from air bubble being pulled through bordered pit: (1) by capillary rupture of an air/water meniscus through pores in the margo when torus aspiration does not occur (margo capillary-seeding, Fig. 1a); (2) by elastic stretching allowing the torus to be pulled out through the pit aperture or through membrane slippage that allows the torus to move off-centre, exposing a portion of the margo in the pit aperture (margo stretch-seeding, Fig. 1b); (3) when the torus and the inner wall of the pit membrane are not perfectly sealed, allowing air bubbles to pass through pores at the edge of the torus (seal capillary-seeding, Fig. 1c); or (4) by rupture in the membrane when low xylem tensions break the fibrils in the margo (margo rupture-seeding, Fig. 1d). Further, an additional mechanism (Fig. 1c) could also occur if the torus structure is not fully impermeable allowing an air bubble to pass through tiny pores (Jansen, Pletsers & Sano 2008; Delzon, personal observations).

To our knowledge, only one study has tested the validity of these different modes of air seeding in gymnosperms (Cochard *et al*. 2009), and concluded that, as for angiosperms, water-stress-induced cavitation occurs through capillary-seeding. Indeed, we clearly rejected the margo rupture-seeding hypothesis by membrane breakage (Fig. 1d) and suggested that the capillary failure should occur when the torus is pinned against the pit aperture (Fig. 1c). However, additional research is needed to assess membrane flexibility (margo stretch-seeding) and torus overlap (seal capillary-seeding) and their respective roles in the air-seeding mechanism. Even though previous studies suggested that torus width and pit chamber depth have implications for margo stretch- and rupture-seeding for four conifer species (Domec *et al*. 2006; Hacke & Jansen 2009), the role of pit membrane structure and function in preventing cavitation is poorly understood. A better insight could be gained by examining interspecific variation of pit membrane structure and function and how it relates to xylem hydraulic safety across a wide range of species spanning along large environmental gradients.

In this paper, we examine whether bordered pit structure and function relates to cavitation resistance across conifer species. For the first time we explore the relationship between cavitation resistance and xylem structure and bordered pit anatomy among 40 conifer species varying widely in cavitation resistance. Cavitation resistance was estimated with the Cavitron technique (Cochard 2002). We studied several bordered pit membrane properties to determine whether the pressure inducing 50% loss of hydraulic conductivity (*P*_50_) is directly linked to mechanical and/or functional properties of the pit membrane. The 40 conifer species sampled provided a wide range of cavitation resistance and xylem anatomy, from the dense and highly resistant wood of *Cupressus* to the soft and least resistant wood of *Metasequoia.* We hypothesized that cavitation resistance is strongly dependent on the capacity of the torus to seal the pit aperture as proposed by Petty (1972). We further hypothesized that such sealing effect should depend on: (1) the degree to which the flexibility of the margo allows the displacement of the torus against the overarching border of the pit; and (2) the torus overlap with the pit aperture (i.e. the ratio of the torus to pit aperture diameter). Based on our results, we discuss the likelihood of the different modes of air-seeding proposed in the literature.

## MATERIALS AND METHODS

### Plant material

During the 2005, 2006 and 2007 spring periods, 40 conifers species representative of a large range of climate dryness were sampled (Appendix S1). Species in six different families were sampled (Farjon 2008): *Araucariaceae* (*n* = 1), *Cupressaceae* (*n* = 12), *Ginkgoaceae* (*n* = 1), *Pinaceae* (*n* = 23), *Podocarpaceae* (*n* = 1) and *Taxaceae* (*n* = 2). Samples were collected in the North America, Europe and Australia from wet to dry habitats in order to have a large range of ecological strategies. Three- to five-year-old branches were sampled in five mature individuals per species in the same stand. Only 40-cm-long straight branches were selected in the upper part of the crown using a telescopic pool-pruner or a slingshot. Immediately after the sampling in the morning, needles were removed and stems were wrapped up with humid paper and conditioned with plastic bags to avoid transpiration. Then, samples were sent to the PIAF laboratory (Clermont-Ferrand, France) and kept refrigerated until measurements.

### Vulnerability to cavitation

Within two weeks after receipt of the samples, xylem cavitation was assessed with the CAVITRON, a centrifuge technique following the procedure described by Cochard (Cochard 2002; Cochard *et al*. 2005). Prior to measurement, all branches were cut under water to a standard length of 27 cm, and bark was removed with a razor blade. Branch samples were not flushed prior measurements as we never observed differences in hydraulic conductivity between flushed and unflushed samples in conifers. Samples were infiltrated with a reference ionic solution of 10 mm 25 KCl and 1 mm CaCl2 in deionized ultrapure water. Centrifugal force was used to generate negative pressure into the xylem and induce cavitation. This method allows to measure xylem conductance under negative pressure. Initially, the maximum conductance of stem (*K*_max_ in m^2^ MPa^−1^ s^−1^) was calculated under low xylem pressures (close to zero) (*P* in MPa). Then, rotation speed of the centrifuge was gradually increased by 0.5 or 1 MPa, to lower xylem pressure. The percentage loss of conductance (PLC) of the stem was determined at each pressure step following the equation:


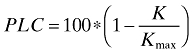
(1)

The relation between *P* and PLC represents the vulnerability curve of the sample. The curve was adjusted with a sigmoid function (Pammenter & Vander Willigen 1998) using the following equation:


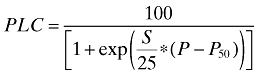
(2)

where *P*_50_ (MPa) is the xylem pressure inducing 50% loss of conductance and *S* (% MPa^−1^) is the slope of the vulnerability curve at the inflexion point. These two parameters were averaged for each species.

### Wood density

To determine whether cavitation resistance varies with wood xylem density (*W*_d_, g cm^−3^) (Hacke *et al*. 2001), the dry mass per fresh volume of the samples was determined according to Archimedes's principle. A 3-cm-long segment was cut from one end of each sample and submerged in water to measure its fresh volume by water displacement using an analytical balance. Then, samples were dried in an oven at 70 °C until constant weight to determine their dry masses.

### Xylem anatomy

One branch out of the five sampled branches per species was randomly selected and used to undertake anatomical measures. Four to five xylem cross sections were cut per sample with a sliding microtome and then fixed on a microscope slide and dyed with safranin. Five photos per section were taken with a digital camera, and analysed with the WinCell® software to measure the lumen area. An average of 500–600 lumens were observed per photo, so an average of 2500–3000 lumens were used to calculate the mean tracheid lumen area for each species (*L*_a_, *µ*m^2^).

Pit membranes were observed with a scanning electron microscope (Fig. 2a). Samples were tangentially cut with a sliding microtome and dehydrated during 48 h in an oven (65 °C). Samples were then coated with gold in high vacuum. Pit aperture diameter (*D*_a_, *µ*m), pit membrane diameter (*D*_m_, *µ*m) and torus diameter (*D*_t_, *µ*m) were measured with ImageJ freeware (http://rsbweb.nih.gov/ij/download.html; see Fig. 2b for parameter descriptions). On average, 20 observations per parameter and species were done.

### Calculation of the valve effect (*V*_ef_)

In addition to structural properties, we characterized some functional properties of the pit membrane. Firstly, to assess the capacity of movement of the torus inside the pit, the margo flexibility index was estimated by comparing the torus and the pit membrane diameter; this index is inversely proportional to the margo strain at aspiration as defined by Hacke *et al*. (2004). Increasing the torus diameter relatively to pit membrane causes a higher margo flexibility. The margo flexibility index (*F*) was estimated as follows:


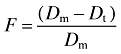
(3)

Following this equation, when *D*_t_ is close to *D*_m_, the length of margo strands is reduced until zero which means that movement of the torus is limited, and so *F* tends to zero. This index is independent of the pit chamber depth and therefore does not take into account the membrane deflected distance from its flat position.

Secondly, to assess the capacity of the torus to seal the pit aperture during an aspiration event, the torus overlap against the pit aperture (*O*) was estimated as:


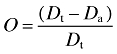
(4)

When *D*_t_ increases in proportion to *D*_a_, the torus is able to seal the pit aperture more efficiently, then *O* tends to 1.

Following our hypothesis, the capacity of the torus to seal the pit aperture during an aspiration should be both dependent of *F* and *O*. The capacity to produce a valve effect (*V*_ef_) was calculated as:


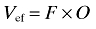
(5)

### Margo capillary-seeding pressure

To test the hypothesis of margo capillary-seeding (Fig. 1a), we measured the biggest pore in the margo. Measurements of margo porosity were made on the most intact regions of the margo. The diameter of the pore was considered as the diameter of the air bubble. Five pit membranes were studied per species. The capillary seeding pressure of the margo (*P_m_* in MPa, opposite of the pressure sustained by the meniscus) was calculated by the following Young-Laplace equation:


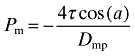
(6)

where *τ* (0.0728 N m^−1^ at 20 °C) is the water surface tension, a (in degrees) is the contact angle between the microfibrills and the meniscus (assumed equal to zero), and *D*_mp_ (*µ*m) is the diameter of the margo pores.

### Statistical analysis

Variations of trait values (*P*_50_ and *S*) among species and families were assessed using a one-way analysis of variance. Correlations between the different variables were tested with a Pearson correlation coefficient (*r*). For non linear relations, data were log transformed and negative values were then converted to positive values. Significant relationships between variables were accepted at *P* < 0.05. Multiple linear regressions were used to determine the combined effect of *F* and *O* on *P_50_*. Statistical analyses were conducted using the SAS software (version 9.2 SAS Institute, Cary, NC, USA).

## RESULTS

### Interspecific variation of vulnerability to cavitation

**Figure 4:**
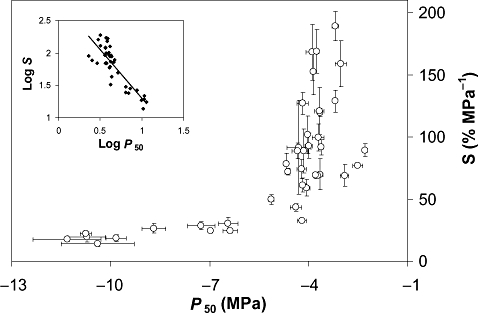
Mean values of vulnerability curve slope (*S*) versus xylem pressure inducing 50% loss in conductance (*P*_50_) measured on 40 species. Error bars represent SE. Log-transformed relationship is shown in the inset.

**Figure 3:**
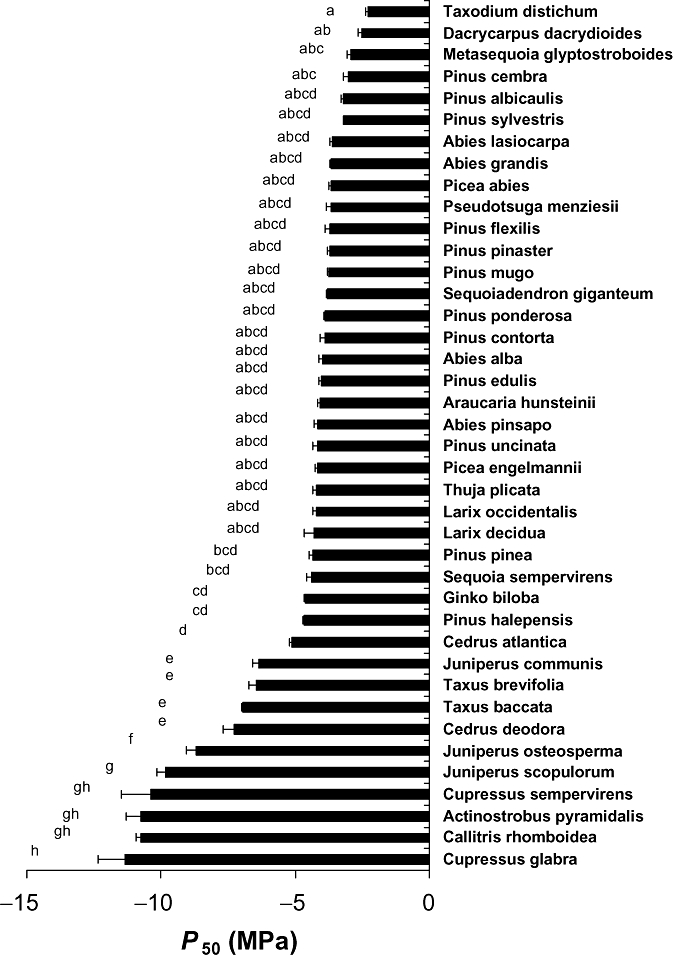
Xylem pressure inducing 50% loss in conductance (*P*_50_) measured in 40 conifer species using the Cavitron technique (*n* = 5 per species). Error bars represent SE. Different letters indicate significant differences between species at *P* < 0.05 (SNK test).

The xylem tension inducing 50% loss in conductivity (*P*_50_) dramatically varied among the species (*F* = 46.17, *P* < 0.0001), ranging from −2.91 MPa for *Metasequoia glyptostroboides* to −11.32 MPa for *Cupressus glabra* (Fig. 3; Appendix S2). The slope of the vulnerability curve (*S*) also significantly differed among species (*F* = 20.01, *P* < 0.0001), ranging between 13.2% MPa^−1^ (*C. sempervirens*) and 189.2% MPa^−1^ (*Pinus albicaulis*). Values of *P*_50_ were significantly different between families (*F* = 28.10, *P* < 0.0001) and within families for *Pinaceae* (*F* = 29.16, *P* < 0.0001) and *Cupressaceae* (*F* = 24.98, *P* = 0.0001). We found a strong correlation between *P*_50_ and *S* (log transformed data, *r* = −0.88, *P* < 0.0001 (Fig. 4), showing that the rate of embolism decreases as cavitation resistance increases and that species with *P*_50_ lower than −6 MPa always embolize very slowly (low *S*).

### Wood density and anatomical traits

Wood density (*W*_d_) varied widely between species, from 0.37 to 0.75 g cm^−3^ for *P. pinaster* and *Taxus brevifolia,* respectively (Appendix S2). Not surprisingly, *W*_d_ was correlated with all xylem anatomical traits measured (Table 1), highlighting the fact that *W*_d_ and tracheids characteristics are mechanically interrelated. The strongest correlations were found with lumen area (*L*_a_) (*r* = −0.68; *P* < 0.0001) and torus diameter (*D*_t_) (*r* = −0.60; *P* = 0.0009), with increases of *L*_a_ and *D*_t_ as wood density decreases. We also found significant relationships between *W*_d_ and pit aperture diameter (*D*_a_) (*r* = −0.58; *P* = 0.0011) and pit membrane diameter (*D*_m_) (*r* = −0.45, *P* = 0.02).

**Table 1:** Correlations among anatomical traits measured on the 40 conifer species. Wood density (*W*_d_); lumen area (*L*_a_); pit membrane diameter (*D*_m_); torus diameter (*D*_t_); pit aperture diameter (*D*_a_)

Variables	*W*_d_ (g cm^−3^)	*L*_a_ (*µ*m^2^)	*D*_m_ (*µ*m)	*D*_t_ (*µ*m)	*D*_a_ (*µ*m)
*W*_d_ (g cm^−3^)		−0.68***	−0.45*	−0.60***	−0.58**
*L*_a_ (*µ*m^2^)	−0.68***		0.43*	0.51**	0.37*
*D*_m_ (*µ*m)	−0.45*	0.43*		0.90***	0.71***
*D*_t_ (*µ*m)	−0.60***	0.51**	0.90***		0.79***
*D*_a_ (*µ*m)	−0.58**	0.37*	0.71***	0.79***	

Values indicate Pearson correlation coefficients (*r*). Bold values indicate significant correlation.

* *P* < 0.05;

** *P* < 0.01;

*** *P* < 0.001.

*L*_a_ was positively and linearly correlated with *D*_t_ (*r* = 0.51; *P* = 0.0083), *D*_m_ (*r* = 0.43; *P* = 0.02) and *D*_a_ (*r* = 0.37; *P* = 0.05) indicating that tracheids with wide lumens allowing high water transport capacity also have wide pits. Moreover, the structural parameters of pit membranes were strongly and positively correlated between one another (Table 1). Large pit membranes were characterized by large *D*_t_, *D*_m_ and *D*_a_.

Concerning the functional properties of the pit membrane, the margo flexibility (*F*) was correlated with *W*_d_ (*r* = 0.57; *P* = 0.0018) and *D*_a_ (*r* = −0.57; *P* = 0.0015). Variations of *F* seemed to be due to *D*_t_ variations rather than to *D*_m_. The torus overlap (*O*) was neither correlated with *W*_d_ and *L*_a_, nor with *D*_m_. Its variability was mainly explained by *D*_a_ variations. It is worth noticing that *L*_a_ was correlated with the pit structural parameters (*D*_m_, *D*_a_ and *D*_t_), but not with the pit functional proprieties (*F*, *O* and *V*_ef_). Therefore, the functional properties of the bordered pit seemed to be independent of the tracheid size.

### Cavitation resistance and xylem structure

**Figure 5:**
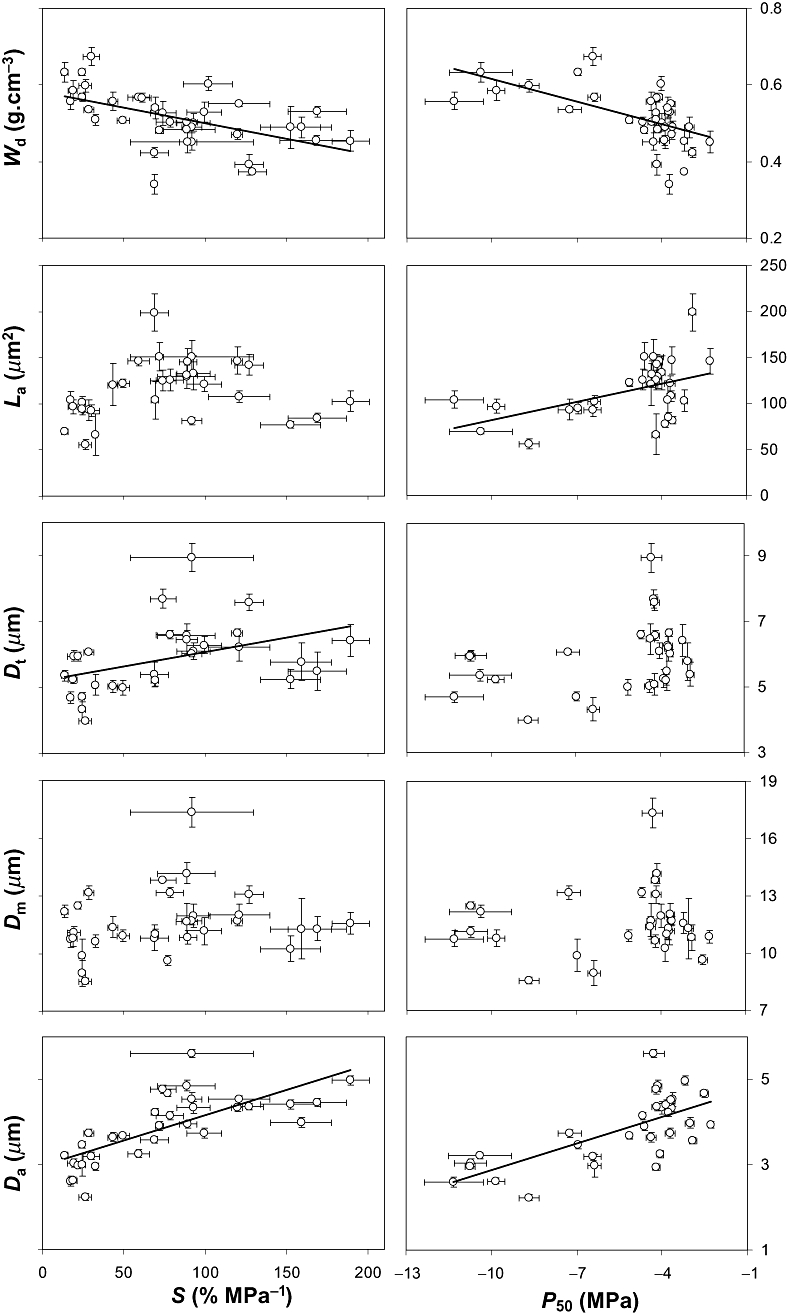
Relationships between wood density (*W*_d_, a), lumen area (*L*_a_, b), torus diameter (*D*_t_, c), pit membrane diameter (*D*_m_, d), pit aperture diameter (*D*_a_, e) and parameters of cavitation resistance (*P*_50_ and *S*). Error bars represent SE. Regression line is indicated when the correlation is significant (*P* < 0.05).

*P*_50_ and *S* were weakly dependent on xylem structural parameters (Table 2 and Fig. 5) but they were strongly correlated with *W*_d_ (*r* = −0.60, *P* = 0.0001 and *r* = −0.53, *P* = 0.0009, respectively). However, this relationship was not significant at the family level or within each family. *P*_50_ was linearly related with *L*_a_ (*r* = 0.48; *P* = 0.006; Fig. 5b), showing that species with a wide tracheid lumen were more vulnerable to cavitation.

**Table 2:** Correlations between anatomical traits (*W*_d_ wood density; *L*_a_ lumen area; *D*_m_ pit membrane diameter; *D*_t_ torus diameter; *D*_a_ pit aperture diameter) and cavitation resistance parameters (*P*_50_ and *S*)

		*W*_d_ (g.cm^–3^)		*L*_a_ (*µ*m^2^)		*D*_m_ (*µ*m)		*D*_t_ (*µ*m)		*D*_a_ (*µ*m)	
Anatomical parameters		Mean	Min	Mean	Max	Mean	Max	Mean	Max	Mean	Max
*P*_50_	*r*	**−0.60**	**−0.56**	**0.48**	**0.52**	0.12	0.05	0.36	**0.39**	**0.73**	**0.73**
	*P*	**0.0001**	**0.0005**	**0.006**	**0.003**	0.52	0.78	0.05	**0.03**	**<0.0001**	**<0.0001**
*S*	*r*	**−0.53**	**−0.54**	0.20	0.16	0.19	0.18	**0.42**	**0.49**	**0.73**	**0.76**
	*P*	**0.0009**	**0.0009**	0.28	0.40	0.31	0.33	**0.02**	**0.006**	**<0.0001**	**<0.0001**

Values indicate Pearson correlation coefficient (*r*) and *P* value (*P*). Bold values indicate significant correlations at *P* < 0.05. Underlined values indicate that correlation was more significant using minimum or maximum values instead of mean.

### Cavitation resistance and bordered pit properties

**Figure 7:**
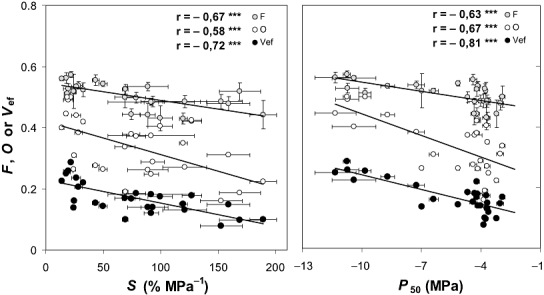
Relationships between functional properties of the bordered pit (torus overlap, *O*, open circles; margo flexibility, *F*, grey circles; valve effect, *V*_ef_, black circles) and cavitation-resistance parameters (*P*_50_ and *S*). Error bars represent SE. Pearson correlation coefficients (*r*) are indicated, *P < 0.05; **P < 0.01; ***P < 0.001.

**Figure 6:**
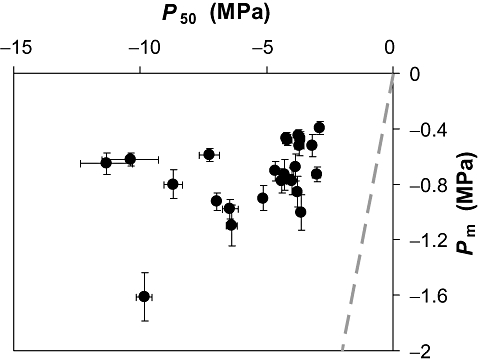
Capillary-seeding pressure of the margo (*P*_m_) versus xylem pressure inducing 50% loss in conductance (*P*_50_). Grey dashed line represent 1:1 relationship.

As shown in Fig. 5, *P*_50_ and *S* variations were also positively related with *D*_a_ (*r* = 0.73; *P* < 0.0001 for both traits) and *D*_t_ (*r* = 0.36; *P* = 0.05 and *r* = 0.42, *P* = 0.02, respectively). However, *P*_50_ and *S* were not related to *D*_m_. Using the maximum values of each parameter (e.g. diameter of the largest pit aperture), only some correlations were weakly enhanced (Table 2). To test the margo capillary-seeding hypothesis, we measured the largest pore inside the margo (between microfibrills) and calculated the pressure required to break the meniscus (*P*_m_). Values of *P_m_* were quite high (close to zero) and systematically greater (less negative) than *P*_50_ values, ranging between −0.4 and −1.6 MPa (Fig. 6). The functional properties of the bordered pit membrane (*F*, *O* and *V*_ef_) were strongly correlated with both *P*_50_ and *S* (Fig. 7). Increasing margo flexibility or torus overlap increased cavitation resistance. Multiple regression analysis showed that the torus/aperture overlap was the best pit functional property explaining P50 variations. Moreover, the steady change in torus/aperture overlap with *P*_50_ and *S* is set by the decrease in pit aperture diameter with increasing cavitation resistance, whereas the torus diameter varies modestly (Fig. 5). Finally, relationships between cavitation resistance traits (*P*_50_, S) and *V*_ef_ were stronger than relationships with *F* and *O* separately.

## DISCUSSION

Inter-specific variability of cavitation resistance in conifers is very large (fourfold), from *C. glabra,* the most resistant species, to *M. glyptostroboides* the least resistant. Such high variability appears to be explained by the functional properties of the bordered pit, specifically the capacity of the torus to seal the pit aperture, which we refer to as valve effect. Species highly resistant to cavitation exhibited a high flexibility of the margo and a large overlap between the torus and the pit aperture, allowing the torus to hermetically seal the pit aperture. In a recent paper, Cochard *et al*. (2009) concluded that margo rupture-seeding was not the determinant of tracheid cavitation and suggested that capillary seeding was the likely mechanism underlying cavitation events in bordered pit membranes. However, the exact location of the capillary failure allowing the entry of air into the tracheid remained enigmatic. As discussed below, our results suggest that such failure occurs at the seal between the torus and the pit aperture.

### Margo capillary-seeding

Previous studies have proposed the margo capillary-seeding as a possible mechanism to explain water-stress induced cavitation (Hacke *et al*. 2004; Cochard 2006; Domec *et al*. 2006). According to this hypothesis the margo elasticity is not large enough to allow the torus to move against the pit border (inner wall) to seal the pit aperture. Subsequent increases of xylem tension cause air-water menisci between margo microfibrils to break allowing air bubbles to embolize adjacent tracheids. The pressure needed to induce this capillary failure depends of the meniscus diameter following the Young-Laplace law. Our results clearly do not support this hypothesis as the capillary seeding pressure of the margo (*P*_m_, calculated with the largest microfibril pore diameter) was systematically and dramatically higher (less negative) than the *P*_50_. *P*_m_ values ranged between −0.4 and −1.1 MPa and were also higher than the xylem air entry pressure (*P*_12_, data not shown), meaning that cavitation never occurred at the pressure inducing capillary failure in the margo. Moreover, Domec *et al*. (2006), for Douglas-fir, estimated the margo deflection pressure (the pressure required to cause the margo to deflect all the way to the pit border) in earlywood and found that the deflection pressure was always higher (less negative) than the capillary seeding pressure of the margo. Therefore, assuming that the margo elasticity is large enough, pit membrane would be deflected and aspirated rather than allowing air seeding though the pores in the margo.

### Margo stretch-seeding

Hacke *et al*. (2004) have suggested that the flexibility of the margo could play a role in the mechanism of cavitation, with more vulnerable species having a more flexible margo. High margo flexibility could allow the torus to be pulled out through the pit aperture or to move off-center, exposing a portion of the margo in the pit aperture. Indeed, it was showed for Douglas-fir that the pressure required to stretch the membrane was similar to the cavitation resistance (*P*_50_), suggesting that the most likely mode of air seeding is by elastic stretching (Domec *et al*. 2006). Contrary to expectations, our results reported a positive correlation between margo flexibility (*F*) and cavitation resistance; the higher the margo flexibility, the more cavitation resistant the species (Fig. 7). A high margo flexibility may facilitate the torus to move towards the pit border and improve the seal between the torus and the pit aperture. Moreover, it's worth noticing that the torus area is always larger than the pit aperture among the 40 conifer species sampled here (51% larger on average), which may prevent the torus from passing through the aperture. However, this size difference does not ensure that the torus might not move off-center. Further investigations are needed to investigate this possibility, particularly by estimating more accurately the margo elasticity (see Hacke *et al*. 2004) and the margo deflected distance in the pit chamber. Indeed, margo elasticity depends on the modulus of elasticity of the microfibril spokes, the strain of the strands, the cross-sectional area of the strands and the total number of strands supporting the torus.

### Seal capillary-seeding

Our data showed that cavitation resistance increased with increasing torus overlap (i.e. increased torus diameter relative to the pit aperture). This result is in agreement with previous findings. Indeed, Hacke & Jansen (2009) reported a weak correlation between the torus overlap and cavitation resistance for three conifer species, resulting in a similar trend to the one we report here (roots tended to have smaller overlap and cavitation resistance than stems). Domec *et al*. (2008) showed that in tall Douglas-fir trees (*Pseudotsuga menziesii*) there was a significant increase in the torus overlap with increasing tree height concurrent with an increase in cavitation resistance. A recent study from Schoonmaker *et al*. (2010) also reported that the torus overlap was significantly larger in open-grown, cavitation resistant individuals compared with understory, less resistant ones. Overall, these results support the hypothesis of seal capillary-seeding, and suggest that the adhesion of the torus to the pit border (i.e. airtightness of the torus – inner wall interface) might be the main determinant of cavitation resistance. Even though the surface tension is presumably sufficiently large to bring the torus into contact with the pit border, adhesive forces between the torus and pit border might be too weak (Comstock & Côté Jr 1968). This could be due to a low margo elasticity preventing the torus from being tightly aspirated. As a tight aspiration is inferred when the imprint of the pit aperture on the torus is clearly visible (Comstock & Côté Jr 1968), detailed studies in different species using scanning electron microscopy are needed to test whether the torus is tightly sealed against the pit border.

Other studies using scanning microscopy have shown that the torus and the inner wall of the pit membrane never appear perfectly smooth such that air seeding could occur through these tiny pores (Kitin *et al*. 2009). Indeed, the presence of warts on the inner wall of the pit membrane can prevent a perfect seal, and thus permit seepage through the pit (Côté Jr 1963). Following the Young-Laplace law, the measured *P*_50_ values correspond to capillary failures of 20 and 100 nm menisci for *C. glabra* and *M. glytpstroboides*, respectively. The presence of these tiny pores has never been investigated and requires therefore more attention. Most recently, the presence of pores extending throughout the entire torus wall has also been observed in *pinus pinaster* trees. These pores were found on a large number of tori and on different individuals, ranging between 20 to 150 nm of diameter (Delzon, personal observations). Such pores were also reported on two conifer species: *Abies sachalinensis* (Sano, Kawakami & Ohtani 1999) and *P. wallichiana* (Jansen *et al*. 2008). Given the size of these pores, air seeding could theoretically occur through the torus by capillary failure. However, current investigations on Cupressaceae species do not seem to confirm this hypothesis and torus thickness was found to be negatively correlated with cavitation resistance (Hacke & Jansen 2009); the thinner the torus, the more cavitation resistant the species.

### The valve effect theory

In light of the results reported here and elsewhere, the valve effect is emerging as the pit functional trait best correlated with cavitation resistance and might explain the air-seeding resistance of bordered pit membranes. This result suggests that pit air-seeding resistance is determined by: (1) a flexible margo allowing the torus to move against the pit border; and (2) a wide torus relatively to the pit aperture to ensure an airtight seal around the pit aperture. A different overlap index was proposed by Hacke *et al*. (2004), corresponding to the variation of the torus diameter relative to the pit membrane and pit aperture diameters. This relative overlap [*D*_t_ − *D*_a_]/[*D*_m_ − *D*_a_] is exactly the fraction of the pit border width that is covered by the torus. However, using this relative index, we found a weaker correlation with both *P*_50_ and slope compared with the torus overlap we have used (data not shown). This finding is consistent with the latter work of Hacke & Jansen (2009), who found no correlation between the relative overlap and *P*_50_, whereas this index seemed to be more correlated with pit hydraulic conductivity.

The comparison of cavitation resistance traits with xylem anatomy and pit functional properties carried out on a broad range of conifer species from six families confirms the key role of the torus-pit membrane apparatus in cavitation resistance. Although our results show that the valve effect, determined by: (1) the deflection capacity of the membrane; and (2) the seal capacity of the torus-pit aperture, is the best predictor of cavitation resistance, additional work is necessary to confirm this theory. In particular, work focusing on the microfibril physical properties and their elastic modulus is required. Here, by a process of elimination, we identified the seal capillary-seeding as the most likely mode of air-seeding in bordered pit of conifer species. However, we cannot reject the hypothesis of torus capillary-seeding and more information on the structural properties of tori is required based on direct observations using transmission electron microscopy. In addition, the mechanical properties of the tracheids (wood density and structure) could play a key role in preventing conduit collapse. For instance, greater tracheid wall thickness would be necessary to resist implosion by negative pressure (Hacke *et al*. 2001). Significant relationships between wood density, lumen area and cavitation resistance were found in the present study and therefore further investigations would be of great interest to estimate the wall implosion pressure and the thickness–to-span ratio.

## SUPPORTING INFORMATION

**Appendix S1.** Study species.

**Appendix S2.** Mean values of parameters of cavitation resistance (*P*_50_ and *S*), wood density (*W*_d_), lumen area (*L*_a_), pit aperture diameter (*D*_a_), torus diameter (*D*_t_) and pit membrane diameter (*D*_m_) for each species. Values in brackets represent standard errors of the mean.
