# Supplementary material for: Mechanism of water-stress induced cavitation in conifers: bordered pit structure and function support the hypothesis of seal capillary-seeding
Source: Plant Cell Environ. 2010 Dec;33(12):2101–11. doi: 10.1111/j.1365-3040.2010.02208.x (PMC3003904; doi:10.1111/j.1365-3040.2010.02208.x)
Supplement: Supplementary file 1 [file pce0033-2101-SD1.docx]

Supplementary materials

**Appendix S1**: Study Species

| **Family** | **Species** | **Range & habitat** | **Sampling location** |
| --- | --- | --- | --- |
| *Araucariaceae* | *Araucaria hunsteinii* K.Schum. | Papua New Guinea | Australia |
| *Cupressaceae* | *Actinostrobus pyramidalis* Miq. | Western Australia | Australia |
|  | *Callitris rhomboidea* Rich. | Western Australia | Australia |
|  | *Cupressus glabra* Sudw. | Arizona, USA | France, Europe |
|  | *Cupressus sempervirens* L. | Mediterranean region | France, Europe |
|  | *Juniperus communis* L. | Europe, Asia and North America | France, Europe |
|  | *Juniperus osteosperma* (Torr.) Little | Southwestern USA | Utah, USA |
|  | *Juniperus scopulorum* Sarg. | Western North America | Montana, USA |
|  | *Thuja plicata* Donn ex D.Don | Northwest America | Idaho, USA |
| *Cupressaceae* (*Taxodiaceae*) | *Metasequoia glyptostroboides* Hu & Cheng | Central China | France, Europe |
|  | *Taxodium distichum* (L.) Rich. | South-eastern North America | France, Europe |
|  | *Sequoia sempervirens* Endl. | California, USA | France, Europe |
|  | *Sequoiadendron giganteum* (Lindl.) J.Buchholz | California, USA | France, Europe |
| *Ginkgoaceae* | *Ginko biloba* L. | Eastern China | France, Europe |
| *Pinaceae* | *Larix decidua* Mill. | Europe | France, Europe |
|  | *Larix occidentalis* Nutt. | Western North America | Idaho, USA |
|  | *Picea abies* ( L.) H.Karst. | Europe | France, Europe |
|  | *Picea engelmannii* Engelm. | Western North America | Montana, USA |
|  | *Pinus albicaulis* Engelm. | Western North America | Montana, USA |
|  | *Pinus cembra* L. | Europe | Austria, Europe |
|  | *Pinus contorta* Douglas | Western North America | Montana, USA |
|  | *Pinus edulis* Engelm. | Western USA | Utah, USA |
|  | *Pinus flexilis* E.James | Western North America | Montana, USA |
|  | *Pinus halepensis* Mill. | Mediterranean region | France, Europe |
|  | *Pinus mugo* Turra | Europe | Austria, Europe |
|  | *Pinus pinaster* Aiton | Mediterranean region | France, Europe |
|  | *Pinus pinea* L. | Mediterranean region | France, Europe |
|  | *Pinus ponderosa* P.Lawson & C.Lawson | Western North America | Montana, USA |
|  | *Pinus sylvestris* L. | Europe and Asia | France, Europe |
|  | *Pinus uncinata* Ramond ex DC. | Europe | France, Europe |
|  | *Pseudotsuga menziesii* (Mirb.) Franco | Western North America | Montana, France |
|  | *Abies alba* Mill. | Europe | France, Europe |
|  | *Abies grandis* Lindl. | Western North America | Idaho, USA |
|  | *Abies lasiocarpa* Endl. | Western North America | Idaho, USA |
|  | *Abies pinsapo* Boiss. | Mediterranean region | France, Europe |
|  | *Cedrus atlantica* G.Manetti | Mediterranean region | France, Europe |
|  | *Cedrus deodara* Loudon | Western Himalayas | France, Europe |
| *Podocarpaceae* | *Dacrycarpus dacrydioides* (A.Rich.) de Laub. | New Zealand | Australia |
| *Taxaceae* | *Taxus baccata* L. | Europe, Africa and Asia | France, Europe |
|  | *Taxus brevifolia* Nutt. | Western North America | Montana, USA |

**Appendix S2**: Mean values of parameters of cavitation resistance (*P*_50_ and *S*), wood density (*W*_d_), lumen area (*L*_a_), pit aperture diameter (*D*_a_), torus diameter (*D*_t_) and pit membrane diameter (*D*_m_) for each species. Values in brackets represent standard errors of the mean.

| **Species** | ***P*_50_** | | ***S*** | | ***W*_d_** | | ***L*_a_** | | ***D*_a_** | | ***D*_t_** | | ***D*_m_** | |
| --- | --- | --- | --- | --- | --- | --- | --- | --- | --- | --- | --- | --- | --- | --- |
| *Abies alba* | -4.00 | (0.11) | 92.87 | (10.21) | 0.502 | (0.023) | 133.42 | (18.49) | 4.34 | (0.14) | 6.10 | (0.24) | 11.97 | (0.60) |
| *Abies grandis* | -3.65 | (0.06) | 119.60 | (3.40) | 0.471 | (0.014) | 146.79 | (14.64) | 4.33 | (0.09) | 6.66 | (0.11) | 11.70 | (0.26) |
| *Abies lasiocarpa* | -3.62 | (0.07) | 91.66 | (6.28) | 0.490 | (0.016) | 81.49 | (3.92) | 4.53 | (0.17) | 6.03 | (0.12) | 11.71 | (0.23) |
| *Abies pinsapo* | -4.15 | (0.14) | 88.64 | (17.55) | 0.483 | (0.009) | 129.25 | (12.68) | 4.85 | (0.13) | 6.58 | (0.15) | 14.19 | (0.52) |
| *Actinostrobus pyramidalis* | -10.73 | (0.57) | 19.50 | (4.05) | - | - | - | - | 3.03 | (0.10) | 5.96 | (0.16) | 11.12 | (0.28) |
| *Araucaria hunsteinii* | -4.07 | (0.11) | 59.03 | (6.63) | 0.568 | (0.004) | 146.28 | (4.97) | 3.25 | (0.09) | - | - | - | - |
| *Callitris rhomboidea* | -10.75 | (0.17) | 22.00 | (1.22) | - | - | - | - | 2.97 | (0.06) | 5.94 | (0.15) | 12.50 | (0.19) |
| *Cedrus atlantica* | -5.14 | (0.08) | 49.63 | (3.81) | 0.508 | (0.008) | 122.43 | (4.30) | 3.68 | (0.06) | 4.99 | (0.23) | 10.92 | (0.30) |
| *Cedrus deodora* | -7.26 | (0.41) | 28.40 | (3.13) | 0.537 | (0.008) | 93.16 | (11.24) | 3.74 | (0.09) | 6.07 | (0.08) | 13.18 | (0.33) |
| *Cupressus glabra* | -11.32 | (1.03) | 17.55 | (1.53) | 0.558 | (0.023) | 104.19 | (9.17) | 2.60 | (0.11) | 4.69 | (0.18) | 10.76 | (0.42) |
| *Cupressus sempervirens* | -10.39 | (1.11) | 13.87 | (1.96) | 0.634 | (0.026) | 69.72 | (2.50) | 3.21 | (0.07) | 5.36 | (0.17) | 12.20 | (0.31) |
| *Dacrycarpus dacrydioides* | -2.52 | (0.16) | 76.80 | (10.92) | - | - | - | - | 4.67 | (0.09) | - | - | 9.64 | (0.25) |
| *Ginko biloba* | -4.62 | (0.04) | 71.99 | (2.57) | 0.481 | (0.007) | 151.14 | (14.91) | 3.90 | (0.10) | - | - | - | - |
| *Juniperus communis* | -6.37 | (0.22) | 24.62 | (1.78) | 0.568 | (0.011) | 101.71 | (6.37) | 2.98 | (0.26) | 4.32 | (0.35) | 8.97 | (0.66) |
| *Juniperus osteosperma* | -8.69 | (0.35) | 26.35 | (3.93) | 0.598 | (0.016) | 55.75 | (5.30) | 2.23 | (0.07) | 3.99 | (0.07) | 8.57 | (0.17) |
| *Juniperus scopulorum* | -9.84 | (0.31) | 18.72 | (2.73) | 0.586 | (0.025) | 96.87 | (7.82) | 2.62 | (0.07) | 5.24 | (0.11) | 10.78 | (0.42) |
| *Larix decidua* | -4.30 | (0.37) | 91.57 | (37.73) | 0.452 | (0.022) | 150.75 | (18.74) | 5.61 | (0.10) | 8.95 | (0.43) | 17.35 | (0.77) |
| *Larix occidentalis* | -4.21 | (0.14) | 74.13 | (7.75) | 0.526 | (0.031) | 125.43 | (10.91) | 4.77 | (0.10) | 7.69 | (0.29) | 13.82 | (0.07) |
| *Metasequoia glyptostroboides* | -2.91 | (0.13) | 68.92 | (8.80) | 0.423 | (0.013) | 199.16 | (20.10) | 3.57 | (0.08) | 5.39 | (0.37) | 10.82 | (0.67) |
| *Picea abies* | -3.66 | (0.09) | 69.72 | (12.46) | - | - | - | - | - | - | - | - | - | - |
| *Picea engelmannii* | -4.18 | (0.09) | 61.23 | (5.61) | 0.567 | (0.013) | - | - | - | - | - | - | - | - |
| *Pinus albicaulis* | -3.19 | (0.10) | 189.24 | (11.61) | 0.454 | (0.026) | 102.89 | (11.49) | 4.98 | (0.11) | 6.42 | (0.48) | 11.58 | (0.56) |
| *Pinus cembra* | -3.02 | (0.17) | 158.87 | (18.57) | 0.490 | (0.028) | - | - | 3.98 | (0.12) | 5.78 | (0.57) | 11.29 | (1.58) |
| *Pinus contorta* | -3.90 | (0.18) | 168.16 | (22.44) | 0.455 | (0.013) | - | - | - | - | - | - | - | - |
| *Pinus edulis* | -4.03 | (0.06) | 101.69 | (15.14) | 0.603 | (0.018) | - | - | - | - | - | - | - | - |
| *Pinus flexilis* | -3.71 | (0.18) | 99.58 | (10.66) | 0.529 | (0.026) | 121.57 | (8.02) | 3.74 | (0.12) | 6.28 | (0.27) | 11.17 | (0.72) |
| *Pinus halepensis* | -4.67 | (0.05) | 78.43 | (8.36) | 0.504 | (0.014) | 125.89 | (11.62) | 4.14 | (0.09) | 6.60 | (0.12) | 13.17 | (0.27) |
| *Pinus mugo* | -3.75 | (0.07) | 168.83 | (17.85) | 0.531 | (0.014) | 84.80 | (5.11) | 4.46 | (0.10) | 5.49 | (0.59) | 11.29 | (0.61) |
| *Pinus pinaster* | -3.73 | (0.07) | 69.00 | (0.50) | 0.341 | (0.027) | - | - | - | - | - | - | - | - |
| *Pinus pinea* | -4.34 | (0.16) | 88.68 | (6.43) | 0.503 | (0.007) | 131.60 | (14.32) | - | - | 6.46 | (0.47) | 11.68 | (0.93) |
| *Pinus ponderosa* | -3.86 | (0.05) | 152.43 | (18.38) | 0.489 | (0.056) | 77.51 | (3.53) | 4.41 | (0.11) | 5.25 | (0.28) | 10.26 | (0.67) |
| *Pinus sylvestris* | -3.20 | (0.02) | 128.79 | (8.71) | 0.374 | - | - | - | - | - | - | - | - | - |
| *Pinus uncinata* | -4.18 | (0.17) | 127.05 | (8.74) | 0.393 | (0.027) | 142.40 | (11.45) | 4.36 | (0.08) | 7.58 | (0.24) | 13.07 | (0.45) |
| *Pseudotsuga menziesii* | -3.68 | (0.15) | 120.77 | (18.93) | 0.551 | (0.007) | 107.90 | (6.10) | 4.53 | (0.08) | 6.21 | (0.43) | 12.02 | (0.56) |
| *Sequoia sempervirens* | -4.38 | (0.17) | 43.33 | (2.92) | 0.557 | (0.024) | 120.71 | (22.88) | 3.64 | (0.11) | 5.03 | (0.20) | 11.38 | (0.52) |
| *Sequoiadendron giganteum* | -3.79 | (0.07) | 69.10 | (2.52) | 0.539 | (0.028) | 103.98 | (20.48) | 4.23 | (0.09) | 5.22 | (0.17) | 11.01 | (0.26) |
| *Taxodium distichum* | -2.29 | (0.07) | 89.36 | (5.35) | 0.451 | (0.028) | 145.79 | (13.97) | 3.94 | (0.08) | - | - | 10.85 | (0.34) |
| *Taxus baccata* | -6.97 | (0.04) | 24.29 | (1.64) | 0.633 | (0.009) | 94.78 | (6.73) | 3.46 | (0.08) | 4.71 | (0.14) | 9.89 | (0.86) |
| *Taxus brevifolia* | -6.44 | (0.30) | 30.23 | (5.16) | 0.675 | (0.023) | 92.57 | (6.57) | 3.19 | (0.09) | - | - | - | - |
| *Thuja plicata* | -4.21 | (0.13) | 32.48 | (1.36) | 0.509 | (0.014) | 66.26 | (22.46) | 2.95 | (0.09) | 5.08 | (0.32) | 10.64 | (0.33) |
